# POWDR: Pathology-Preserving Outpainting with Wavelet Diffusion for 3D MRI

**DOI:** 10.3390/diagnostics16152385

**Published:** 2026-07-29

**Authors:** Fei Tan, Ashok Vardhan Addala, Bruno Astuto Arouche Nunes, Xucheng Zhu, Ravi Soni

**Affiliations:** 1 GE HealthCare, San Ramon, CA 94583, USA; 2 GE HealthCare, Menlo Park, CA 94025, USA

**Keywords:** diffusion models, outpainting, 3D MRI, pathology preservation, medical image synthesis

## Abstract

**Background:** Medical imaging datasets often suffer from class imbalance and limited availability of pathology-rich cases, which limit the performance of machine learning models for segmentation, classification, and vision–language tasks. We propose POWDR, a pathology-preserving outpainting framework for 3D MRI that uses real pathological regions as conditioning evidence while generating anatomically plausible surrounding tissue. **Methods:** Our approach leverages wavelet-domain conditioning to enhance high-frequency detail and mitigate blurring common in latent diffusion models. We introduce a random connected mask training strategy to reduce conditioning-induced collapse and improve diversity outside the lesion. POWDR is evaluated on brain MRI using BraTS datasets and extended to knee MRI to assess applicability beyond brain imaging. **Results:** Quantitative metrics (FID, MS-SSIM, LPIPS) were used to assess image realism. Random connected mask training improved diversity, reducing cosine similarity from 0.9947 to 0.9580 and increasing KL divergence from 0.00026 to 0.01494. To validate pathology preservation, we compared lesion overlap, volume, intensity, and morphology. For downstream segmentation, nnU-Net performance improved from 0.6992 to 0.7137 Dice after augmentation with 50 synthetic cases, representing a modest but statistically significant improvement (paired *t*-test, *p* = 0.016). Tissue volume analysis showed no significant differences for CSF and GM compared to real images, while WM volume was lower in synthetic images. **Conclusions:** POWDR provides a framework for generating diverse, pathology-preserving synthetic MRI data. The results suggest potential utility for data augmentation while maintaining clinically relevant lesion characteristics.

## 1. Introduction

The development of medical imaging analysis methodologies using deep learning is hindered by two major challenges: the scarcity of pathological data and pronounced class imbalance. Acquiring large-scale, diverse datasets is resource-intensive, and many pathological conditions occur at low prevalence, limiting the availability of representative samples. These constraints impede the training of robust and generalizable models for segmentation, classification, and related clinical imaging tasks. Consequently, developing strategies to augment the availability of pathology-rich images without incurring additional acquisition costs is critical. Ideally, such augmentation should preserve real pathological findings and their annotations while increasing the diversity of the surrounding anatomical context.

Generative modeling has become an increasingly practical tool for alleviating data scarcity and class imbalance in medical imaging, especially when pathological cases are rare [1]. However, most synthesis or completion approaches either generate pathology implicitly [2], replace pathology with pseudo-healthy tissue [3], or perform super-resolution across slices [4]. In this work, we focus on a complementary setting: pathology-preserving outpainting, where the real lesion region is retained and the surrounding anatomy is synthesized to form full 3D volumes. This enables clinically grounded augmentation by varying the non-pathological context while keeping real pathology fixed and interpretable.

A central technical challenge in outpainting is preserving fine anatomical structure, particularly boundaries and high-frequency detail, while generating large unseen regions. Latent diffusion models [5] are efficient but can blur high-frequency content due to compression in the autoencoder latent space [6,7], which is undesirable for realistic anatomical synthesis. Wavelet-based diffusion [8,9] mitigates this by modeling multi-scale frequency subbands directly, allowing the generative process to represent sharp structural detail more faithfully. While wavelet diffusion has been explored for paired synthesis tasks in 3D medical imaging, its utility for pathology-preserving outpainting has not been systematically studied.

Related work in 3D diffusion has largely focused on inpainting: filling missing or pathological interior regions given surrounding context, often to reconstruct pseudo-healthy tissue. Durrer et al. [10] evaluate diffusion-based 3D healthy tissue inpainting and compare 2D, pseudo-3D, 3D, latent, and wavelet variants, emphasizing consistency within the filled region; fastWDM3D [11] further improves sampling efficiency for wavelet diffusion inpainting. Ferreira et al. [12] apply 3D wavelet diffusion to BraTS tasks including inpainting, while Friedrich et al. [13] apply conditional wavelet diffusion to missing-modality generation. In contrast to these inpainting and modality-translation objectives, the present study targets lesion-preserved outpainting, a setting in which real pathology is used as conditioning evidence while the surrounding anatomical context is generated.

In this work, we introduce POWDR, a pathology-preserving outpainting framework for 3D MRI built on conditioned wavelet diffusion. POWDR synthesizes the surrounding anatomy outside the conditioned region and maintains the lesion appearance without explicitly copying voxels. A key methodological component is our random connected mask training strategy, which mitigates conditioning collapse and enables variable-region, multi-direction outpainting from arbitrary contexts. Together, these design choices provide a practical and controllable mechanism for generating diverse, pathology-preserving 3D volumes for augmentation when pathological scans are scarce.

The main contributions of the study are as follows:

1. We formulate pathology-preserving outpainting as a clinically motivated augmentation problem for 3D MRI, distinct from pseudo-healthy inpainting, unconditional pathology generation, and super-resolution.
2. We propose POWDR, a conditioned wavelet diffusion framework for generating anatomically plausible surrounding tissue while preserving lesion-conditioned information.
3. We introduce random connected mask training to mitigate conditioning collapse and improve diversity in the generated anatomical context.
4. We validate the approach on brain MRI and knee MRI, evaluating image realism, pathology preservation, diversity, and downstream segmentation utility.

## 2. Materials and Methods

### 2.1. Network Design

#### 2.1.1. Training

**Figure 1:**
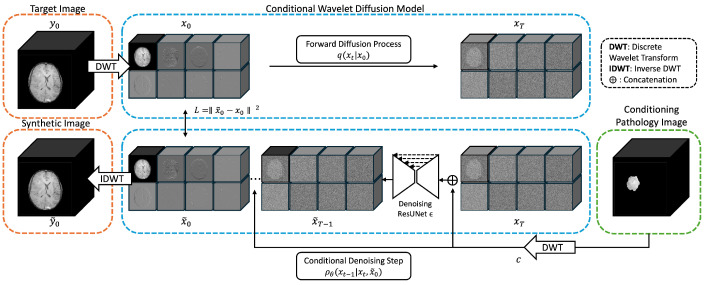
Architecture of POWDR: a pathology-preserving outpainting framework using a conditioned wavelet diffusion model for 3D MRI. During training, the target MRI volume is decomposed into eight wavelet subbands using a 3D Haar transform, forming the diffusion target. The conditioning image is generated either from a tumor-masked image or a randomly connected mask region multiplied by the source image. The noisy target wavelet coefficients and conditioning coefficients are concatenated and processed by an attention-based ResUNet. During inference, only lesion-masked conditioning images are used. The network does not directly copy lesion voxels into the output; instead, pathology preservation is learned through conditional generation, and the final image is reconstructed using inverse wavelet transforms.

Our architecture (Figure 1) builds upon the conditional wavelet diffusion framework [13] with several enhancements for 3D medical image synthesis. In the forward diffusion process, the original 3D MRI volume $y_{0}\in R^{H\times W\times D}$ is decomposed using a discrete wavelet transform (DWT) with a Haar kernel [14] into $x_{0}\in R^{8\times \frac{H}{2}\times \frac{W}{2}\times \frac{D}{2}}$, which contains eight subbands:

(1) $$x_{0}=DWT\left(y_{0}\right)=\left\{LLL,LLH,LHL,LHH,HLL,HLH,HHL,HHH\right\}$$

where LLL,LLH,LHL,LHH,HLL,HLH,HHL, and HHH represent low- (*L*) and high-frequency (*H*) components along the three spatial axes. Gaussian noise ϵ∼N(0,I) is progressively added according to a linear diffusion schedule with $\beta_{t}$ ranging from 0.0001 to 0.02, where t∈{1,…,T} denotes the diffusion step and T=1000 in this study. The parameter $\beta_{t}$ controls the noise variance added at step *t*, $\alpha_{t}=1-\beta_{t}$ denotes the retained signal fraction at that step, and ${\bar{\alpha }}_{t}=\prod_{i=1}^{t}\alpha_{i}$ denotes the cumulative signal retention from step 1 to step *t*. The forward diffusion process produces the noisy wavelet-domain image $x_{t}$:

(2) $$x_{t}=\sqrt{{\bar{\alpha }}_{t}}x_{0}+\sqrt{\left(1-{\bar{\alpha }}_{t}\right)}\epsilon .$$

For conditional denoising, we employ a 3D attention-based ResUNet [15] architecture. The conditioning image is also transformed into wavelet subbands and denoted as *c*. At each denoising step, *c* is concatenated with the noisy target subbands $x_{t}$ as $\left[x_{t},c\right]$, forming 16 input channels. The conditioning image during training can be either a masked pathology region or a randomly masked region, as detailed in the Dataset section. The network estimates ${\tilde{x}}_{0}$, the predicted clean wavelet-domain target corresponding to $x_{0}$, from the noisy input $x_{t}$ at diffusion step *t*; during sampling, this prediction is used to compute the reverse update toward ${\tilde{x}}_{t-1}$. The predicted ${\tilde{x}}_{0}$ contains eight channels, and the mean squared error (MSE) in the wavelet domain is adopted as the loss function:

(3) $$L=MSE_{wavelet}={∥x_{0}-{\tilde{x}}_{0}∥}^{2}$$

Importantly, the diffusion process and denoising network operate only on the target image channels: the conditioning channels *c* are provided as fixed inputs at every step, but not denoised, and are not explicitly injected or copied into the final output. Thus, pathology preservation is learned through conditional generation rather than enforced by voxel replacement.

Training was iteration-based rather than epoch-based. The model was trained for 2,000,000 iterations with a batch size of 1. Neither early stopping nor a validation dataset was used. Other key training parameters included a dropout rate of 0.1, 1000 diffusion time steps, a ResUNet base channel size of 64 with channel multipliers of [1, 2, 2, 4, 4], the AdamW optimizer [16], and a learning rate of $1\times 10^{-5}$. All experiments were conducted on an NVIDIA H100 GPU with 80 GB of VRAM. The architecture and detailed training parameters are summarized in Appendix A, Table A1.

#### 2.1.2. Sampling

During inference, masked pathology region conditioning is achieved by element-wise multiplication of the pathology mask with the corresponding intensity volume (e.g., the tumor mask with the brain image or the cartilage mask with the knee image). This approach ensures that pathological regions are preserved while enabling synthesis of surrounding anatomy. We apply 1000 denoising steps following the Denoising Diffusion Probabilistic Model (DDPM) sampling strategy [17]. The generated sample, initially represented in wavelet space, is then reconstructed into the image domain using the Inverse Discrete Wavelet Transform (IDWT).

(4) $${\tilde{y}}_{0}=IDWT\left({\tilde{x}}_{0}\right)$$

#### 2.1.3. Baseline Conditional Latent Diffusion Network

To the best of our knowledge, there are currently no established 3D diffusion models specifically designed for pathology-preserving MRI outpainting. Therefore, we implemented two conditional latent diffusion baselines using the most widely adopted latent diffusion framework in medical image generation. To ensure a comprehensive comparison, we evaluated both continuous latent representations (VAE-cLDM) and discrete latent representations (VQGAN-cLDM), which constitute the dominant design choices in modern latent diffusion models. These baselines were trained using the same conditioning inputs, training data, and sampling protocol as POWDR.

In the absence of established 3D outpainting networks, we implemented two variants of conditional latent diffusion (cLDM) [5] as baseline models. The framework uses a two-stage design. In Stage 1, a latent autoencoder compresses full-resolution brain MRI volumes into a 4-channel latent representation. In Stage 2, a conditional diffusion model generates synthetic latent volumes conditioned on masked tumor regions.

For latent compression, we evaluated a Variational Autoencoder (VAE) [18] and a Vector Quantized GAN (VQGAN) [19]. The VAE employs a 3D encoder–decoder with progressive downsampling (channel multipliers: 1, 2, 4, 8; base channels: 32), producing four latent channels for mean and log variance. The VQGAN uses the same backbone but replaces the continuous latent variables with a discrete codebook of 8192 embeddings (dimension 4), using nearest-neighbor quantization and a commitment loss. When adversarial training is enabled, a 3D PatchGAN discriminator [20] provides patch-level realism feedback. Both autoencoder variants are optimized with mean squared reconstruction loss.

For conditional generation, the Stage 2 diffusion model operates on the frozen latent space. A 3D U-Net [21] with 128 base channels and the same multi-resolution structure as the autoencoder serves as the denoiser. Conditioning is provided by concatenating lesion-masked latent volumes with the noisy latent input. The model follows 1000 diffusion steps, cosine noise scheduling, and an L2 loss targeting the clean latent signal. The diffusion model was trained for 1500 epochs to match the number of steps of cWDM, using AdamW with a $1\times 10^{-4}$ learning rate.

### 2.2. Datasets

#### 2.2.1. Brain MRI Dataset

We used the BraTS 2023 dataset for POWDR model training, comprising 1251 cases from the T1n MRI sequence, and the BraTS 2024 Additional dataset for POWDR synthetic image generation and downstream segmentation testing [22,23,24,25]. In this study, the BraTS T1n sequence and the corresponding tumor annotations were used for pathology conditioning and evaluation. The conditioning tumor mask was obtained by binarizing the BraTS ground-truth segmentation, with all voxels labeled greater than zero treated as tumor foreground; this included voxels belonging to the whole tumor (WT), tumor core (TC), and enhancing tumor (ET) regions. Although the present experiments focused on T1n imaging, the same framework can be extended to other BraTS MRI contrasts, including T2w, T1 contrast-enhanced, and T2-FLAIR. The BraTS 2024 Additional cases were divided into two non-overlapping subsets: 100 cases were used for synthetic data generation and augmentation experiments, while an independent set of 171 unseen cases was reserved exclusively for downstream segmentation and pathology-preservation evaluation. No patient overlap existed between the augmentation cohort and the segmentation test cohort.

The default training strategy used the masked tumor region as the conditioning input. Specifically, the tumor mask was multiplied element-wise with the corresponding MRI volume to retain only the pathology-containing region. To increase conditioning diversity, we introduced an additional training strategy that used random connected masks multiplied by the image volume as training conditions. In this strategy, six connected random masks were generated and multiplied by the image volume to form conditioning inputs. The mask sizes were sampled from the empirical tumor-volume distribution in the training set to approximate realistic lesion extents. All images were intensity-normalized to the 1st-99th percentile, cropped to a 224×224×155 matrix size, and maintained at 1 mm isotropic resolution. Identical preprocessing was applied to all training, augmentation, and testing datasets. During sampling for both training scenarios, the tumor-masked intensity volume was used for conditioning, so sampling remained pathology-conditioned.

#### 2.2.2. Knee MRI Dataset

We further validated our approach on a knee MRI dataset comprising 768 3D volumes collected across multiple scanners and two distinct MRI sequences. The first subset is a public dataset acquired using Double Echo Steady State (DESS) sequences on Siemens scanners (Erlangen, Germany), originally described by [26] and used in a segmentation challenge [27]. This subset includes 176 MRI volumes from 88 patients, with manual 3D segmentation masks for four tissue compartments—patellar, tibial, and femoral cartilage, as well as the menisci—generated by a single expert reader [27].

The second subset is an internal dataset of 592 studies from 275 unique patients. These volumes were acquired using CUBE, a high-resolution 3D Fast Spin Echo (FSE) sequence developed by GE Healthcare, and previously utilized by [28] for anomaly and object detection. Scans were obtained on five 3-T MRI scanners (GE Healthcare, Waukesha, WI, USA) between 2006 and 2018, spanning multiple scanner models and sites.

All knee MRI volumes were preprocessed to ensure consistency: cropped to a common field of view (144×144×80 mm), resized to 288×288×160 voxels, and resampled to 0.5 mm isotropic resolution. Cartilage and meniscus masks were combined and dilated by 3 pixels using a spherical kernel to account for thin structures and then multiplied element-wise by the image volume to form the condition. Both healthy and pathological cases were included in training. During sampling, 20 additional cases with meniscus tear pathology, none of which had been used during model training, served as pathology-conditioned inputs. This setup demonstrates that the model can leverage both healthy and pathological images during training while enabling pathology-conditioned synthesis—a capability particularly valuable when pathological cases are scarce.

### 2.3. Evaluation

#### 2.3.1. Quantitative Metrics

We report standard generative metrics: Fréchet Inception Distance (FID) [29], which measures how closely the distribution of generated images matches that of real images; Multi-Scale Structural Similarity (MS-SSIM) [30], which evaluates the preservation of anatomical details; and Learned Perceptual Image Patch Similarity (LPIPS) [31], which assesses how visually similar two images appear to a human observer. FID was computed using Med3D network [32] features for medical relevance (details are discussed in Appendix B). For brain MRI, FID, MS-SSIM, and LPIPS were computed using the 100 synthesized images generated from the BraTS 2024 augmentation cohort and their corresponding real images; these synthesized images were also used in the downstream nnU-Net augmentation experiments. MS-SSIM and LPIPS were calculated separately inside and outside the pathology region to assess pathology preservation and anatomical diversity. For brain MRI, the inside-pathology region was defined by the binary BraTS tumor mask with ground-truth label >0; for knee MRI, the inside-pathology region was defined by the conditioning mask used for meniscus pathology synthesis.

To quantify sample diversity from repeated sampling of the same conditioning input, we computed pixel-wise mean and standard deviation across samples to assess spatial variation in generated outputs, as well as pairwise similarity metrics across all unique sample pairs. Cosine similarity was calculated by flattening each 3D volume into a 1D vector and computing the normalized dot product between sample pairs, where lower values indicate greater diversity. KL divergence was computed by binning intensity values into 50-bin histograms to form probability distributions for each sample, followed by calculating the Kullback–Leibler divergence between all pairs using the formula: $\mathrm{KL}\left(P\|Q\right)=\sum_{x}P\left(x\right)\log\frac{P(x)}{Q(x)}$. Higher KL values indicate more diverse intensity distributions. For *N* samples, this resulted in N(N−1)/2 pairwise comparisons, and the mean across all pairs was reported as the final diversity metric.

#### 2.3.2. Clinical Relevance Analysis

We evaluate downstream utility via whole tumor segmentation using nnU-Net [33]. The baseline uses 1251 BraTS 2023 cases + 100 BraTS 2024 Additional cases. Synthetic augmentation experiments add 10, 50, or 100 generated images conditioned on real pathology extracted from the same 100 BraTS 2024 cases. This training set selection ensures that performance variation across models is introduced by the synthetic POWDR images rather than by additional real pathologies in the training set. nnU-Net is trained for 1000 epochs with default parameters, and Dice scores are computed on 171 unseen test cases from BraTS 2024.

Additionally, we assess anatomical plausibility by comparing brain tissue volumes (cerebrospinal fluid: CSF, gray matter: GM, and white matter: WM) between real and synthetic images using FSL FAST segmentation [34] (version 6.0.7.19). We analyze 100 synthetic and 100 real cases, reporting volume statistics.

#### 2.3.3. Pathology Preservation Analysis

To assess whether the conditioned pathology was preserved beyond image-level similarity metrics, we performed paired pathology analysis between each real image and its corresponding POWDR-generated image. For brain MRI, paired synthetic images were generated from the 171 reserved BraTS 2024 test cases using the corresponding tumor-masked conditioning inputs. The original and synthetic images were then processed using the best-performing nnU-Net segmentation model to obtain tumor masks for both images. Pathology preservation was quantified using Dice overlap between the real and synthetic tumor masks, tumor volume, mean lesion intensity, and shape perimeter extracted from the segmented lesion mask. These measurements were selected to evaluate spatial overlap, lesion size, intensity consistency, and boundary-related morphology, respectively.

#### 2.3.4. Statistical Analysis

Unless otherwise specified, quantitative values are reported as mean ± standard deviation. Statistical significance was defined as p<0.05. For downstream segmentation, Dice scores were compared on the same 171 held-out test cases using a paired *t*-test between the baseline model and the synthetic-augmentation model. For pathology preservation analysis, normality of paired measurements was assessed using the Shapiro–Wilk test. When normality was rejected, paired Wilcoxon signed-rank testing was used to compare real and synthetic measurements.

## 3. Results

### 3.1. Brain MRI Results

#### 3.1.1. Qualitative Results

**Figure 2:**
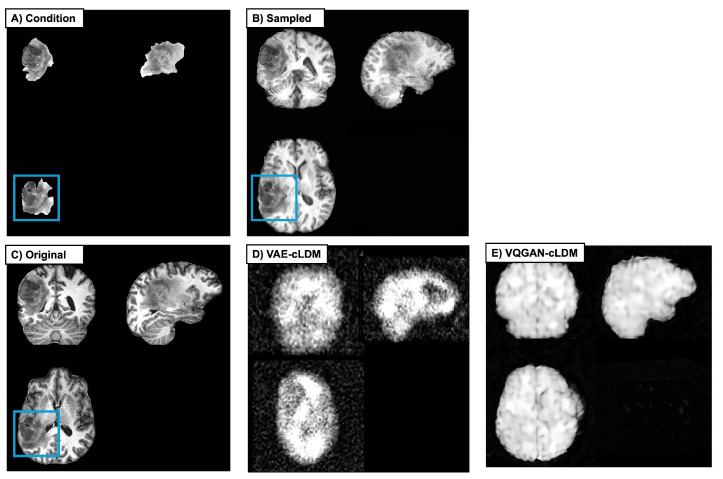
Qualitative example of brain MRI outpainting. Shown are (**A**) the input condition (masked tumor), (**B**) the synthetic image generated by POWDR, and (**C**) the original image from which the tumor was extracted. Highlighted boxes indicate tumor regions preserved during synthesis. (**D**) Synthetic image generated using a VAE-cLDM outpainting model. (**E**) Synthetic image generated using a VQGAN-cLDM outpainting model.

Figure 2 illustrates three-plane views of the conditioning pathology image, the sampled synthetic image, and the original image from which the conditioning pathology was extracted. In addition to our proposed cWDM results, we include conditional latent diffusion (cLDM) baselines using VAE and VQGAN autoencoders. The VAE-cLDM baseline did not converge to coherent brain anatomy, producing outputs without recognizable brain structure. The VQGAN-cLDM baseline yielded a coarse brain-shaped appearance, but the anatomical details were blurred and structurally inaccurate, and the conditioned lesion was not clearly recognizable, indicating weaker pathology preservation and poorer anatomical fidelity than cWDM.

#### 3.1.2. Quantitative Results

We evaluate similarity to real images using FID, MS-SSIM, and LPIPS (Table 1). Because the VAE-cLDM baseline failed to learn meaningful brain structure, we report quantitative comparisons for VQGAN-cLDM only. Metrics were computed on 100 synthetic–real pairs from the BraTS 2024 augmentation cohort. cWDM achieves a substantially lower FID (0.0042) than VQGAN-cLDM (0.1681), indicating that cWDM samples more closely match the distribution of real scans under the same feature extractor. Within pathology regions, cWDM shows near-perfect structural agreement (MS-SSIM-in = 0.9998 ± 0.0003) and very low perceptual distance (LPIPS-in = 0.0030 ± 0.0017), consistent with substantial pathology preservation. In contrast, VQGAN-cLDM exhibits reduced lesion fidelity (MS-SSIM-in = 0.9860 ± 0.0146, LPIPS-in = 0.0368 ± 0.0164), aligning with the qualitative observation that lesions are less recognizable. Outside pathology regions, cWDM shows better anatomical consistency with the corresponding real images (MS-SSIM-out = 0.8195 ± 0.0517, LPIPS-out = 0.1484 ± 0.0311), whereas VQGAN-cLDM shows larger perceptual deviation (MS-SSIM-out = 0.7380 ± 0.0580, LPIPS-out = 0.3995 ± 0.0561), reflecting blurrier and less anatomically consistent synthesis. Additionally, VAE and VQGAN reconstruction-integrity analyses are included in Appendix C.

**Figure 3:**
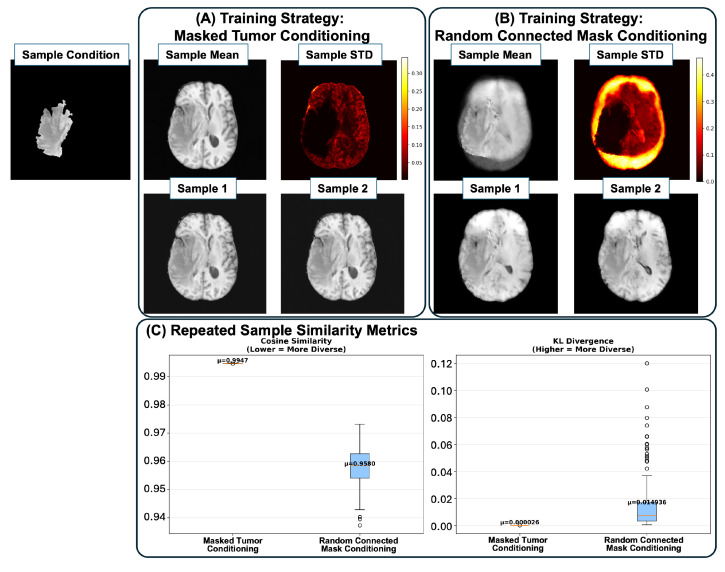
Sampling diversity under different training strategies. (**A**,**B**) Mean, standard deviation, and example outputs from 20-case repeated sampling using (**A**) masked tumor conditioning and (**B**) random connected mask conditioning. (**C**) Cosine similarity and KL divergence quantify improvements in diversity.

To assess diversity, we performed repeated sampling from the same conditioning input. Figure 3A shows that when trained with masked tumor conditioning, generated samples exhibit small variation, an issue commonly observed in strongly conditioned diffusion models. The mean sample across 20 repetitions appears nearly identical, and the standard deviation is low, indicating limited sampling diversity. To address this, we introduce random connected masks during training. Figure 3B shows a substantial increase in standard deviation across repeated samples in regions outside the pathology, while remaining minimal within the conditioned pathology area, when trained with random connected masked region conditioning. The two example outputs further illustrate greater variability in anatomical structures such as CSF cavities and brain boundaries. Figure 3C further shows that strategy B substantially increases diversity: cosine similarity between pairwise samples decreases from 0.9947 ± 0.00003 to 0.9580 ± 0.0066, and KL divergence increases from 0.00026 to 0.01494.

This diversity enhancement is important for downstream tasks. With tumor-mask conditioning (Strategy A), the maximum augmentation equals the number of unique conditions *N*, doubling the dataset at best. In contrast, random-mask training (Strategy B) enables the generation of nN samples with greater contextual variation, where *n* is user-defined and can be optimized to provide flexible augmentation without overwhelming the real data in downstream tasks.

#### 3.1.3. Clinical Relevance Results

**Figure 4:**
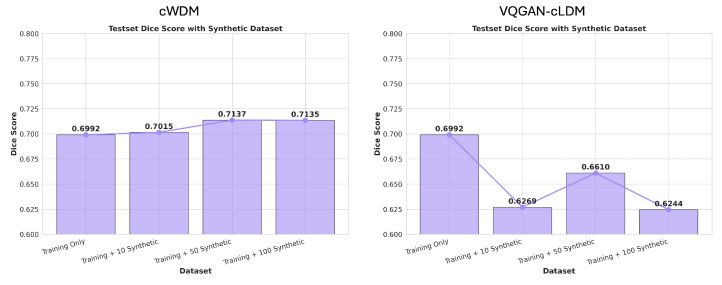
Impact of synthetic augmentation on tumor segmentation. Dice scores for nnU-Net segmentation under varying numbers of synthetic cases (10, 50, 100) compared to baseline.

To evaluate downstream utility, we trained nnU-Net using identical real training data with and without synthetic augmentation. To isolate the effect of POWDR as data augmentation, synthetic images were generated from the same 100 BraTS 2024 cases used in segmentation training. Dice scores improved from 0.6992 ± 0.3009 (baseline) to 0.7015 ± 0.2955 with 10 synthetic cases, plateauing at 0.7137 ± 0.2868 for 50 cases and 0.7135 ± 0.2866 for 100 cases (Figure 4). The best performance was obtained using 50 synthetic cases, increasing mean Dice from 0.6992 to 0.7137. Paired statistical testing across 171 independent test cases showed that this modest improvement was statistically significant (paired *t*-test, *t* = −2.43, *p* = 0.016). Although the absolute gain was small, the results suggest that POWDR-generated images may provide useful additional variation for segmentation model training.

**Figure 5:**
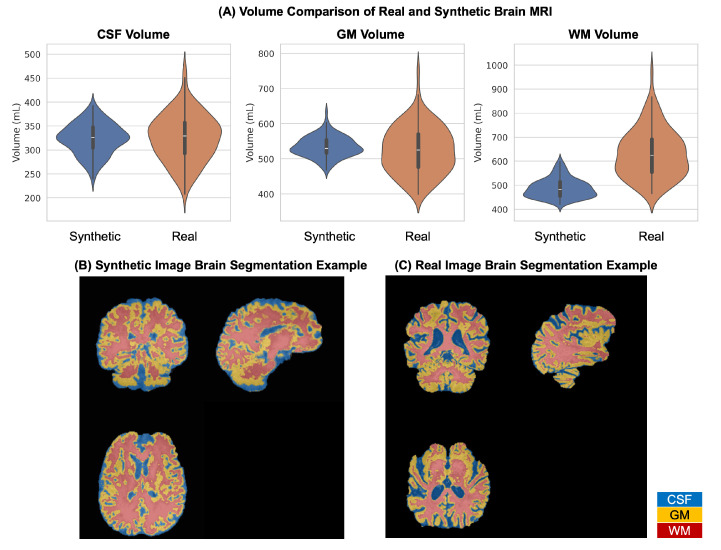
Comparison of brain tissue volumes between real and synthetic images. Volumes of CSF, gray matter, and white matter segmented using FSL FAST for 100 synthetic and 100 real cases.

For anatomical credibility, we performed brain tissue segmentation using the FSL FAST algorithm. As shown in Figure 5, CSF volumes (Synthetic: 323.428 ± 32.001 mL, Real: 325.397 ± 49.228 mL; *p* = 0.738) and GM volumes (Synthetic: 534.475 ± 29.312 mL, Real: 524.283 ± 66.766 mL; *p* = 0.164) showed no significant difference between synthetic and real images. WM volume was lower in synthetic images than in real images (synthetic: 487.882 ± 39.418 mL; real: 631.305 ± 100.213 mL; *p* = $2.57\times 10^{-29}$), likely because tumor presence affected tissue segmentation.

#### 3.1.4. Pathology Preservation Analysis Results

To quantitatively evaluate pathology preservation, we compared lesion overlap, volume, intensity, and shape characteristics between real images and synthetic images generated from the same conditioning pathology. Because Shapiro–Wilk testing rejected normality for the evaluated metrics (p<0.05), paired Wilcoxon signed-rank testing was used for statistical analysis. In the absence of formal radiologist review, we used the best-performing nnU-Net segmentation model to obtain tumor masks from both real and synthetic images and performed paired comparisons of lesion Dice overlap, tumor volume, lesion intensity, and shape perimeter.

The 171 reserved test cases were used to generate paired synthetic images. Both the original and synthetic images were then processed using the nnU-Net model trained with real data plus 50 POWDR-generated synthetic cases, which achieved the best downstream segmentation performance. The generated lesions showed substantial spatial agreement with the corresponding real lesions, with a mean Dice score of 0.741±0.254 for the combined tumor region. Mean lesion intensity was highly similar between real and synthetic images (0.682 vs. 0.680, p=0.549), and shape perimeter showed no significant difference (213.57 mm vs. 213.54 mm, p=0.283). Tumor volume was slightly larger in synthetic images than in real images (57.20 cm^3^ vs. 53.56 cm^3^, p=0.0014), indicating a statistically significant but relatively modest volume shift.

Overall, these results suggest that POWDR preserves key lesion characteristics, including spatial overlap, intensity distribution, and boundary-related shape features. Although the tumor volume metric exhibited a statistically significant difference, the magnitude of the change was small relative to the inter-patient variability in tumor appearance. This difference may reflect both residual generative variation and uncertainty introduced by automatic segmentation. Therefore, the pathology preservation results should be interpreted as evidence of substantial, but not perfect, preservation of lesion morphology and texture.

### 3.2. Knee MRI Results

**Figure 6:**
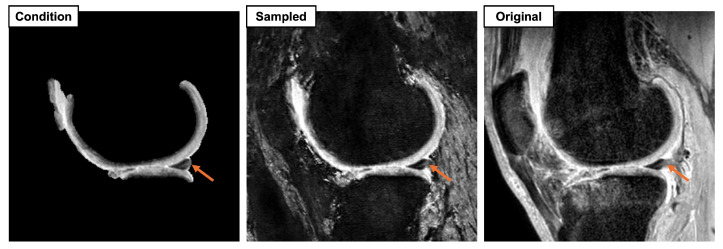
Qualitative results for knee MRI outpainting. The model preserves the meniscus tear conditioning region (orange arrows) while generating surrounding anatomy, demonstrating the framework’s applicability beyond brain MRI.

Figure 6 shows qualitative results for 3D knee MRI outpainting. Despite the larger matrix size and longer training time, the model preserves the meniscus tear conditioning region while generating plausible surrounding anatomy. This demonstrates the model’s potential applicability beyond brain MRI and highlights its possible use for pathology augmentation in low-prevalence conditions. We note that the contrast of the sampled images differs noticeably from the original images, likely because of heterogeneity in MR image contrast and the scanner vendors represented in the diffusion model’s training set.

As shown in Table 2, for knee outpainting, cWDM achieves a 3D FID of 0.0014, suggesting that the generated volumes are close to the distribution of real knee MRI under the same 3D feature extractor. Region-specific perceptual and structural metrics further suggest effective preservation of the conditioned pathology region with variable surrounding synthesis. Inside the conditioned pathology mask, LPIPS is low (0.0037 ± 0.0016) and MS-SSIM is near perfect (0.9997 ± 0.0002), consistent with strong fidelity to the provided pathology cue. Outside the conditioned region, LPIPS increases (0.3675 ± 0.0585) and MS-SSIM decreases (0.5826 ± 0.0741), reflecting appearance variation in the synthesized anatomy. Overall, these results suggest that cWDM preserves the conditioned pathology region while introducing variable context in the non-conditioned regions, which is desirable for augmentation-oriented outpainting.

The knee MRI experiment evaluated the feasibility of transferring POWDR to a second anatomical domain. Although preservation of the meniscus tear remained visually convincing, noticeable contrast differences between the generated and original images were observed. We hypothesize that these differences reflect heterogeneity in the scanner vendors and MRI sequences represented in the training set.

## 4. Discussion

This study investigated pathology-preserving outpainting as an augmentation-oriented image synthesis problem for 3D MRI. The central motivation differs from that of conventional unconditional image generation, pseudo-healthy inpainting, or super-resolution. Instead of synthesizing pathology de novo or replacing abnormal tissue with normal-appearing anatomy, POWDR uses a real pathological region as conditioning evidence and generates the surrounding anatomical context. This design is intended to retain clinically meaningful lesion information and its associated annotation while increasing the diversity of non-pathological anatomy available for model training.

The quantitative and qualitative results suggest that conditioned wavelet diffusion is well suited to this setting. Compared with conditional latent diffusion baselines, POWDR produced more coherent brain anatomy, lower FID, and better region-specific similarity within the conditioned pathology region. This finding is consistent with the motivation for operating in the wavelet domain: high-frequency anatomical boundaries and tissue interfaces may be degraded by aggressive latent compression, whereas wavelet subbands preserve explicit multi-scale structural information. At the same time, the baseline comparison should be interpreted in the context of the task. To our knowledge, there is no established public 3D diffusion framework specifically designed for pathology-preserving MRI outpainting, and the VAE-cLDM and VQGAN-cLDM models were selected as task-matched latent diffusion baselines rather than as an exhaustive comparison against all possible generative architectures.

The random connected mask strategy addressed an important practical issue in conditional generation. When training used only tumor-masked conditions, repeated sampling from the same condition produced highly similar outputs, limiting the value of the model for augmentation. Random connected mask training increased variability outside the conditioned pathology while maintaining low variation within the pathology region. This behavior is desirable for augmentation because multiple samples generated from the same lesion should not simply duplicate the same anatomical context. The observed reduction in cosine similarity and increase in KL divergence support the role of this strategy in improving contextual diversity, although future work should further quantify the optimal number and composition of synthetic samples for downstream training.

Pathology preservation was evaluated using lesion overlap, volume, intensity, and boundary-related morphology rather than relying only on global image realism metrics. The generated brain lesions showed substantial spatial agreement with the corresponding real lesions, and lesion intensity and shape perimeter were not significantly different between real and synthetic images. Tumor volume was slightly larger in synthetic images, with a statistically significant but relatively modest shift. This finding suggests that POWDR preserves important lesion characteristics but does not produce a perfect replica of the original pathology. Some discrepancy may arise from residual generative variation, from the fact that the lesion is learned through conditioning rather than copied into the output, and from uncertainty introduced by automatic tumor segmentation. These results support substantial pathology preservation, but they also motivate future expert review and more detailed lesion-level validation.

The downstream nnU-Net experiment provides preliminary evidence that POWDR-generated images can be useful for model development. Adding 50 synthetic cases improved mean Dice from 0.6992 to 0.7137 on 171 independent test cases, and the paired test indicated statistical significance. However, the absolute improvement was small and the variability across cases was large, reflecting the heterogeneity of tumor size and appearance in the test cohort. Therefore, this result should be interpreted as evidence of potential augmentation utility rather than a demonstrated clinical performance gain. Additional experiments across model architectures, tasks, and external datasets will be needed to determine when pathology-preserving outpainting yields practically meaningful improvements.

While the brain MRI experiments provided the quantitative evaluation, the knee MRI experiment was designed to assess feasibility and cross-anatomical transferability. POWDR preserved meniscus tear conditioning regions while generating surrounding knee anatomy, indicating that the method is not restricted to a single organ system. From an augmentation perspective, this image synthesis technique could increase the representation of low-prevalence diseases without requiring additional lesion annotation. Nevertheless, the knee MRI experiment also highlights current limitations. Visible contrast differences were observed between generated and original knee images. These differences likely reflect scanner, vendor, and sequence heterogeneity in the knee dataset, particularly because the data combined DESS and CUBE acquisitions. Future extensions should investigate scanner-aware conditioning, sequence-specific training, or harmonization strategies to improve cross-protocol consistency before broader clinical use.

Several limitations should be acknowledged. First, no formal radiologist reader study was performed to further evaluate pathology preservation. The current validation relies on objective image metrics, lesion-level measurements, tissue volume analysis, and downstream segmentation, which provide reproducible evidence but do not replace expert assessment of diagnostic realism. Second, the observed tumor volume difference should be interpreted cautiously because it may reflect not only residual generative variation but also uncertainty from the automatic segmentation algorithm and differences in the generated surrounding background. Third, validation was limited to brain tumor MRI and a knee MRI cohort with 20 meniscus tear cases; additional studies across institutions, scanners, sequences, anatomical regions, and disease types are needed to assess generalizability.

Overall, POWDR should be viewed as a controllable synthetic data generation strategy for pathology-preserving augmentation rather than as a stand-alone diagnostic tool. Its main value lies in generating plausible anatomical context around real pathology while retaining a direct link to observed lesion information. This makes the framework potentially useful for training and stress-testing segmentation, classification, and multimodal imaging models in settings where pathology-rich data are limited, while also underscoring the need for further clinical, statistical, and privacy validation.

## 5. Conclusions

In this work, we introduced POWDR, a pathology-preserving outpainting framework for 3D MRI based on a conditioned wavelet diffusion model. To our knowledge, this is among the first approaches to address MRI outpainting while explicitly preserving real pathological regions. We evaluated POWDR using conventional generative metrics, including FID, MS-SSIM, and LPIPS, as well as repeated-sampling diversity analysis. In addition, we performed quantitative pathology-preservation analyses across independent test cases, demonstrating preservation of lesion overlap, intensity, and shape features. Augmentation with POWDR-generated images produced a modest but statistically significant improvement in nnU-Net segmentation performance on an independent test cohort, suggesting potential downstream utility. Beyond brain MRI, the knee MRI experiment showed that the framework may be applicable to additional anatomical domains. Collectively, these results suggest that POWDR can generate diverse, pathology-preserving synthetic MRI data and may serve as a practical strategy for alleviating data scarcity and class imbalance in 3D medical imaging.

## Figures and Tables

**Table 1:** Quantitative evaluation of synthetic brain MRI realism and pathology preservation using 100 synthetic–real pairs from the BraTS 2024 augmentation cohort. Values in parentheses indicate standard deviation. FID was computed at the cohort level; MS-SSIM and LPIPS were computed inside and outside the pathology region.

Metrics	cWDM	VQGAN-cLDM	Real vs. Real
FID ↓	0.0042	0.1681	0.0005
MS-SSIM—inside pathology ↑	0.9998 ± 0.0003	0.9860 ± 0.0146	-
MS-SSIM—outside pathology	0.8195 ± 0.0517	0.7380 ± 0.0580	-
LPIPS—inside pathology ↓	0.0030 ± 0.0017	0.0368 ± 0.0164	-
LPIPS—outside pathology	0.1484 ± 0.0311	0.3995 ± 0.0561	-

↑ Higher values indicate better performance; ↓ Lower values indicate better performance.

**Table 2:** Quantitative evaluation of synthetic knee MRI realism and pathology preservation. Values in parentheses indicate standard deviation. FID was computed at the cohort level; MS-SSIM and LPIPS were computed inside and outside the conditioned pathology region.

Metrics	cWDM
FID ↓	0.0014
MS-SSIM—inside pathology ↑	0.9997 ± 0.0002
MS-SSIM—outside pathology	0.5826 ± 0.0741
LPIPS—inside pathology ↓	0.0037 ± 0.0016
LPIPS—outside pathology	0.3675 ± 0.0585

↑ Higher values indicate better performance; ↓ Lower values indicate better performance.

## Data Availability

The BraTS data used in this study are openly available through the BraTS Challenges at https://www.synapse.org/Synapse:syn53708126/wiki/626320 (accessed on 1 August 2025). The OAI knee MRI data are available through OAI at https://nda.nih.gov/oai (accessed on 10 April 2025). The internal GE HealthCare knee MRI data are not publicly available due to privacy and institutional restrictions.

## Abbreviations

The following abbreviations are used in this manuscript:

POWDR	Pathology-Preserving Outpainting with Wavelet Diffusion for 3D MRI
MRI	Magnetic Resonance Imaging
IDWT	Inverse Discrete Wavelet Transform
cWDM	Conditional Wavelet Diffusion Model
cLDM	Conditional Latent Diffusion Model
DDPM	Denoising Diffusion Probabilistic Model
DDIM	Denoising Diffusion Implicit Model
ResUNet	Residual U-Net
U-Net	Convolutional Networks for Biomedical Image Segmentation
VAE	Variational Autoencoder
VQGAN	Vector-Quantized Generative Adversarial Network
GAN	Generative Adversarial Network
PatchGAN	Patch-based Generative Adversarial Network
FID	Fréchet Inception Distance
MS-SSIM	Multi-Scale Structural Similarity Index Measure
LPIPS	Learned Perceptual Image Patch Similarity
KL	Kullback–Leibler
CSF	Cerebrospinal Fluid
GM	Gray Matter
WM	White Matter
MSE	Mean Squared Error
MAE	Mean Absolute Error
PSNR	Peak Signal-to-Noise Ratio
nnU-Net	No-New-U-Net
BraTS	Brain Tumor Segmentation Challenge
FSL	FMRIB Software Library
FAST	FMRIB’s Automated Segmentation Tool
DESS	Double Echo Steady State
FSE	Fast Spin Echo
OAI	Osteoarthritis Initiative

## Appendix A. Additional Implementation Details

**Table A1:** Architecture and training parameters for POWDR.

Parameter	Value
Backbone	3D ResUNet
Resolution levels	5
Blocks per level	2
Input size	224×224×155 (brain), 288×288×160 (knee)
Input channels	16 (8 noisy target wavelet channels + 8 conditioning wavelet channels)
Feature channels	base channels = 64, scaled by [1,2,2,4,4] at each resolution level
Output channels	8
Diffusion steps	1000
Noise schedule	Linear, $\beta_{start}=1\times 10^{-4},\beta_{end}=0.02$
Loss function	MSE loss
Optimizer	AdamW
Learning rate	$1\times 10^{-5}$
Batch size	1
Training steps	2,000,000
Early stopping	None
Dropout rate	0.1
Hardware	1 NVIDIA H100 GPU
Framework	PyTorch 2.3.1

## Appendix B. 3D FID Computation Using MedicalNet (Med3D) Features

To evaluate distributional similarity between generated and real 3D volumes, we compute FID in a 3D feature space rather than using the conventional 2D Inception network. Specifically, each 3D volume is passed through a 3D-ResNet pretrained on diverse medical datasets (MedicalNet/Med3D), and we extract deep feature embeddings from the network’s final pooling layer (global average pooled activations). We then fit multivariate Gaussians to the embeddings of real and generated volumes and compute the Fréchet distance between them using the standard FID formulation [29]. Using a 3D medical feature extractor better reflects volumetric anatomy and avoids slice-wise aggregation artifacts that can arise in 2D pipelines.

Because FID depends on the embedding network, preprocessing, and domain, absolute FID values are not directly comparable across studies that use different feature extractors (e.g., 2D Inception vs 3D MedicalNet [32]) or different normalization/cropping conventions. We therefore use FID primarily for relative comparisons across methods under identical evaluation settings (same feature network, preprocessing, and sample size).

## Appendix C. Autoencoder Reconstruction Quality (VAE vs. VQGAN)

To assess whether the poor qualitative performance of latent diffusion baselines is attributable to insufficient latent compression quality, we evaluate reconstruction fidelity for both the VAE and VQGAN autoencoders on held-out validation volumes. Both autoencoders achieve strong voxel-level reconstruction accuracy, with PSNR = 34.68 dB for the VAE and PSNR = 32.33 dB for the VQGAN at epoch 99 (Table A2). Corresponding error metrics are low (VAE: MSE = 0.000363, MAE = 0.006589; VQGAN: MSE = 0.000621, MAE = 0.008572). Both models compress volumes into the same latent tensor shape (4×28×28×20), corresponding to a 128× compression ratio.

Despite similarly high reconstruction fidelity, the downstream cLDM training behavior differs substantially: the VAE-based cLDM fails to converge to coherent brain anatomy, while the VQGAN-based cLDM produces a coarse brain-like structure but with blurred anatomy and degraded lesion appearance. These results indicate that autoencoder reconstruction quality alone is insufficient to guarantee a diffusion-friendly latent space under conditioning; the limiting factor is instead the stability and expressiveness of the latent generative modeling process when learning conditional synthesis.

**Table A2:** Reconstruction quality.

Metrics	VAE	VQGAN
MSE	0.000363	0.000621
MAE	0.006589	0.008572
PSNR	34.68 dB	32.33 dB
